# Effects of ultrasound on the component, anti-inflammatory activity of adlay seed polyphenols and the mechanism of alleviating colitis

**DOI:** 10.1016/j.ultsonch.2026.108143

**Published:** 2026-10-29

**Authors:** Yue Wu, Changsheng Guo, Xiaoqiang Li, Jianjun Cheng, Qingfeng Ban

**Affiliations:** College of Food Science, Northeast Agricultural University, Harbin, Heilongjiang 150030, China

**Keywords:** Adlay seed polyphenols, Ultrasonication, Colitis, Gut microbiota, PI3K-Akt pathway

## Abstract

Adlay (*Coix lacryma-jobi L.*) seeds are rich in polyphenols with diverse bioactivities. Ultrasound-assisted extraction (UAE) was used to prepare adlay seed polyphenols extract (ASP), and the effects of ultrasonic intensity on the composition and *in vitro* anti-inflammatory activity of ASP were evaluated by UPLC-QTOF-MS and a cellular inflammation model. The anti-colitis mechanism of ASP obtained at the optimal ultrasonic power was further validated in dextran sulfate sodium (DSS)-induced colitis mice. Fourteen major phenolic compounds were identified in ASP. Moderate-intensity ultrasound at 300 W significantly increased the phenolic constituents of ASP by 61.01%, mainly kaempferol, ferulic acid, and coniferyl alcohol, and enhanced its anti-inflammatory bioactivity. In DSS-induced mice, ASP intervention preserved intestinal barrier integrity, suppressed pro-inflammatory cytokines, and promoted anti-inflammatory cytokine secretion. 16S rRNA sequencing further showed that ASP restored gut microbial homeostasis by decreasing pro-inflammatory *Escherichia-Shigella* (57.14%) and *Bacteroides* (45.83%) and enriching beneficial *Lachnospiraceae_NK4A136_group* (68.49%), supporting recovery of basal metabolic function and intestinal epithelial repair. Transcriptomic profiling and qPCR validation confirmed that ASP alleviated colitis by suppressing aberrant activation of the PI3K-Akt signaling pathway. Collectively, UAE enhanced the anti-inflammatory bioactivity of ASP, providing a basis for its development and application in colitis intervention.

## Graphical abstract

**Figure:**
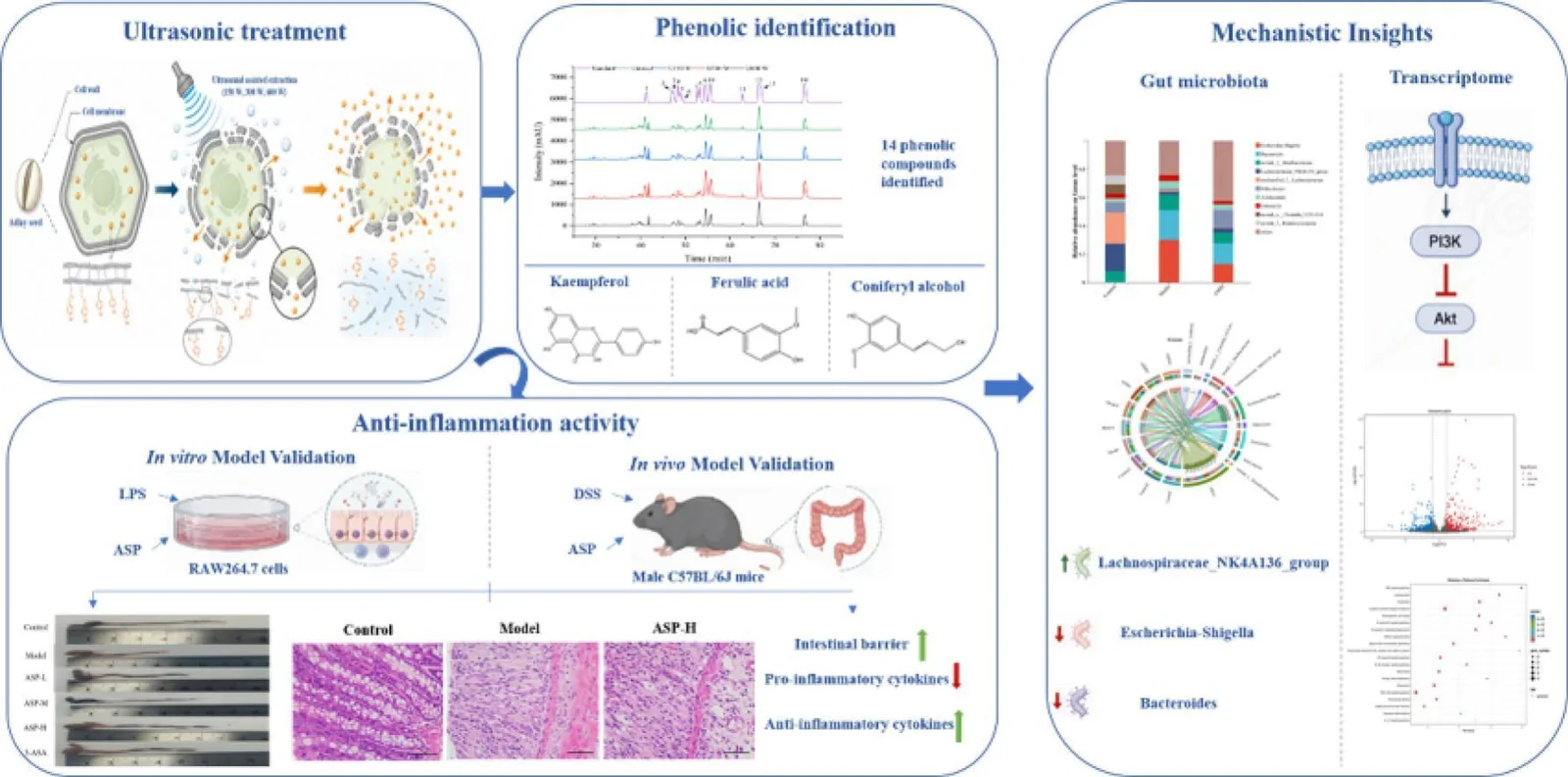

## 1 Introduction

Inflammatory bowel disease (IBD) represents a group of chronic, idiopathic, hard‑to‑treat inflammatory disorders targeting the gastrointestinal tract. In recent decades, incidence rate of IBD has steadily increased worldwide [1], [2]. As a worldwide pervasive intractable illness, IBD is marked by relapsing intestinal inflammation, mucosal injury and malabsorption, which greatly compromise patients’ quality of life. Furthermore, the aging IBD patient population is accompanied by an elevated risk of secondary complications, including intestinal fibrosis, intestinal obstruction, and even colorectal cancer, which further increases the global disease burden and socioeconomic medical pressure [3]. Current clinical management of IBD primarily rely on synthetic drugs such as antibiotics, corticosteroids, sulfasalazine, and 5-aminosalicylic acid [4]. However, these therapies are often ineffective in curing the disease, and each drug has limitations, including potential induction of psychiatric side effects, poor tolerance, possible nephrotoxicity, and various inflammatory complications [5], [6]. Clinically, a substantial proportion of IBD patients require lifelong medication for symptom control, and an optimal, safe, and curative therapeutic strategy for IBD remains absent. Lack of safety, affordability, and comparative effectiveness of integrated drugs urgently requires new, safe, and green intervention strategies for comparative prevention and treatment of IBD.

Natural green interventions based on plant-derived bioactive compounds have emerged as a research hotspot for adjuvant IBD therapy due to their high biosafety, low toxicity, and multi-target regulatory properties [7]. The predominant green intervention substances for IBD include plant polyphenols, flavonoids, terpenoids, and polysaccharides, which exert intestinal protective and anti-inflammatory effects via two core mechanisms: gut microbiota modulation and inflammatory signaling pathway regulation. Host-microbiota dynamic crosstalk and inflammatory signaling pathway activation constitute two independent but mutually coordinated core regulatory mechanisms underlying green IBD intervention [8]. Patients with IBD commonly exhibit profound gut microbiota dysbiosis, which is manifested as marked depletion of beneficial commensal bacteria and proliferation of pathogenic taxa. This microbial imbalance can disrupt intestinal mucosal barrier, causing persistent chronic intestinal inflammation and accelerating progression of IBD [9]. Aside from the modulatory effects on intestinal microbes, mounting studies indicate that dysregulated PI3K‑AKT signaling represents a critical molecular event contributing to IBD pathogenesis. Under inflammatory excitation, PI3K phosphorylates and activates AKT, triggering downstream effector molecules including Bcl-2 and mTOR, which regulate cell proliferation and apoptosis, while also affecting inflammatory cascade and exacerbating damage caused by intestinal inflammation [10]. Natural green bioactive compounds (such as plant polyphenols, polysaccharides, and alkaloids) can have a dual protective effect on gut: they efficiently improve composition of gut microbiota, enrich beneficial bacterial communities, inhibit growth of pathogenic microorganisms, restore gut microbiota balance, inhibit abnormal activation of PI3K-AKT signaling pathway, block inflammatory signal transmission, reduce release of pro-inflammatory factors, alleviate intestinal inflammatory reactions, and achieve intestinal mucosal repair [7]. In addition, with the rapid advancement of omics technologies, the combined application of transcriptomics and 16S rRNA gene sequencing enables high-throughput, systematic detection of differential gene expression and microbial community variation post-treatment, providing a powerful technical approach to elucidate the multi-target and multi-pathway mechanisms of green IBD interventions [8].

Among diverse natural phytochemicals, grain polyphenols exhibit superior advantages for green IBD intervention. As food-derived natural bioactive components, grain polyphenols possess high biosafety, excellent biocompatibility, low toxicity, and good long-term edible adaptability, making them more suitable for the long-term adjuvant intervention of chronic IBD compared with synthetic drugs and other plant-derived active substances [11]. Moreover, grain polyphenols have multi-target regulatory effects, consistently regulating gut microbiota and repairing integrity of intestinal barrier, suppressing inflammatory signaling pathways. They have broad potential for application in prevention and treatment of intestinal inflammatory diseases, and their potential value is worth evaluating [12]. Adlay seed (*Coix lacryma-jobi* L.), a typical Gramineae plant with homologous medicinal and edible properties, is widely cultivated in Southeast Asia, with China ranking among the world’s most important producing regions [13]. Extract of adlay seed in modern pharmacology regulates inflammatory response and oxidative stress, thereby reducing damage to gastric mucosa [14]. Analysis results of phytochemistry show that adlay seed contains abundant phenolic compounds [15]. Polyphenolic constituents derived from this grain (ASP) display a broad spectrum of biological functions, among which anti‑inflammatory and antioxidant effects stand out. Studies have shown that it can alleviate various inflammatory damages, inhibit the progression of colon tumors, improve osteoporosis, regulate intestinal immunity, down-regulate intestinal inflammatory factors, and regulate the intestinal microbiota [16], [17]. It is expected to be used in the intervention of inflammatory bowel diseases. However, polyphenols in grains primarily exist in bound forms, predominantly associated with cell wall polysaccharides [18], with only a small fraction readily bioavailable. It is necessary to use enrichment methods to support improvement of polyphenol content in grains, that is, their biological activity, and enhance their bioavailability. This holds significant importance for the development and application of adlay seed and its polyphenolic compounds. Traditional extraction approaches exhibit inherent drawbacks including poor extraction performance and high energy input, thereby limiting the scalable production and practical utilization of high-quality ASP [19]. Ultrasound, as a processing technique, offers several advantages. Compared to thermal treatment, it operates at lower temperatures, helping preserve heat-sensitive nutrients and bioactive compounds such as polyphenols, which are prone to degradation at high temperatures [20]. Additionally, ultrasound consumes less energy and generates fewer greenhouse gases, making it an environmentally friendly alternative [21]. Furthermore, ultrasonic cavitation produces intense shear forces and micro-jet impacts that disrupt plant cell wall structures, causing the dissociation of bound polyphenols linked to cell wall polysaccharides and promoting the release of low-molecular-weight phenolic compounds with superior biological activity, thereby enhancing their antioxidant and anti-inflammatory properties [22], [23]. Li et al. found that the ultrasound treatment affected the content of polyphenols in honeysuckle, which increasing the levels of chlorogenic acid and luteoloside, thereby enhancing their antioxidant activity [24]. Ultrasound-induced mechanical vibration and cavitation reactions serve as the core driving force for this phenomenon, enabling the efficient release of active polyphenols that bind tightly to raw material matrices. Zhang et al. investigated the mechanism by which ultrasound activates polyphenols in highland barley and further demonstrated that appropriate ultrasonic treatment conditions can optimize the molecular structure of polyphenols, reduce structural damage, and thereby significantly enhance their free radical scavenging capacity and biological activity [25]. However, systematic studies on the relationship between ultrasound treatment and ASP’s anti-inflammatory activity and its ability to alleviate colitis remain limited.

Hence, this study aims to evaluating the effects of ultrasonic treatment on the phenolic content and bioactivity of ASP. Study will also investigate efficacy of ASP in alleviating colitis, ASP has dual effects in improving intestinal inflammation and regulating composition of intestinal microbiota. Using RNA seq and PCR analysis methods to elucidate molecular working mechanism and signaling pathways of anti colitis activity. This provides new insights into how ASP can alleviate colitis and lays a scientific foundation for potential use of new functional foods.

## 2 Materials and methods

### 2.1 Materials

The adlay seed material was supplied by Shanxi Dongfangliang Biotechnology Co., Ltd. (Shanxi, China). DSS with a molecular weight range of 36 000–50 000  Da was obtained from MP Biomedicals (California, USA). Antibodies specific for ZO‑1, Occludin, and Claudin‑1 were procured from Abcam Corporation (Shanghai, China). Reagents used for qPCR experiments were purchased from Bio‑Rad Laboratories (Shanghai, China). Remaining chemicals of analytical grade were obtained from commercial vendors.

### 2.2 Extraction of adlay seed polyphenols

Adlay seed powder was defatted and mixed with pre-cooled 80% acetone at the material − liquor rate of 1:5 (w/v). Cell ultrasonic fragmentation was implemented via a JY92-IIDN cell disruptor (Ningbo Scientz Biotechnology Co., China). A constant ultrasonic frequency of 20 kHz and a 3-s cyclic interval were maintained during the entire treatment process. The extraction was performed at 150 W, 300 W, and 600 W power for 30 min under an ice bath. After extraction, samples were placed in a 4 ℃ environment for centrifugation operation, with a speed set to 1684 × g and a duration of 15 min, and supernatant was collected. Use same steps as above to extract remaining material twice more. The supernatant was concentrated by spinning and freeze-dried. The resulting lyophilized powder was stored at −20 ℃ protected from light for subsequent analysis [26].

### 2.3 Identification of polyphenol composition

This study employed a high-resolution mass spectrometer to identify the phenolic compound composition of adlay seed (UPLC-QTOF-MS, AB SCIEX, CA, USA). Mass spectrometer operates in negative ion research mode, with a capillary potential difference set at −4500 V. Nitrogen was used as the drying gas at a flow rate of 35 mL/min, with a drying temperature of 450 ℃. Collision energy was set at −40 V, nebulizer pressure at 50 psi, and the mass-to-charge ratio range at 100–1000 *m*/*z*.

Subsequently, samples from adlay seed polyphenols extracted at varying ultrasonic power levels were analyzed using a reversedphase HPLC system (Thermo Fisher Co., Germany) and a C18 chromatographic column (4.6 mm × 250 mm, 5 μm). Injection with 0.2 mL/min, 10 μL and 280 nm wavelength. The fluid phase mainly consists of two parts, namely (A) acetonitrile plus 0.1% formic acid and (B) an aqueous solution containing 0.1% formic acid. The solvent A increased linearly from 0% to 17% at 0–15 min, from 17% to 28% at 15–28 min, from 28% to 60% at 28–50 min, from 60% to 70% at 50–65 min, from 70% to 80% at 65–75 min, from 80% to 0% at 75–80 min, and from 80% to 0% at 80–90 min. At 80–90 min solvent A remained at 0%. Quantification was achieved using external reference standards, and summarize milligrams of phenolic compound per 100 g of sample [27].

### 2.4 Cells culture

RAW264.7 cell lines needs to be cultured in a humid environment with a temperature of 37 ℃ and 5% CO_2_. Prior to experiments, all cell lines underwent a minimum of three passages to ensure phenotypic stability [28].

### 2.5 Cytotoxicity measurements

Use Cell Counting Kit-8 (CCK-8) to evaluate cytotoxicity of ASP. RAW264,7 cells (5 × 10^4^ cells/well) were cultured for 24 h. Add ASP (0–1500 μg/mL, DMEM) under requirements of standardized cultivation for 24 h. Process by adding 10% CCK-8 100 μL reagent to each well and incubating for 1 h. Measure absorbance at 450 nm using an enzyme marker (SpectraMax i3x, Molecular Devices, USA). Estimation of cell survival rate is given to percentage of absorbance of treated cells compared to untreated cells [28].

### 2.6 Intracellular ROS level

RAW264.7 cells (1.0 × 10^5^ cells/well) were treated with ASP (250, 500, or 1000 μg/mL) for 24 h. To induce inflammation, cells were subsequently added to 1 μM LPS for 24h, and DCFH-DA (10 μM) was added to culture medium at 37 °C for 20 min. The fluorescent intensity was calculated using an enzyme marker (SpectraMax i3x, Molecular Devices, USA) with an excitation of 488 nm and emission at 525 nm. All results have been normalized and expressed as relatively values compared to untreated normal controls. LDH activity in the supernatant was determined according to the kit’s official instructions [29].

### 2.7 Antioxidant enzyme activities

RAW264.7 macrophages were seeded at 2.5 × 10^5^ cells/mL and maintained in culture for 24 h prior to treatment. A 24-hour ASP intervention was then performed at concentrations of 250, 500 and 1000 μg/mL, with untreated cells serving as the negative control. Upon completion of pre-treatment, 1 μM LPS was added to all groups and incubated for 24 h to trigger intracellular inflammatory responses. Cellular oxidative stress indicators, including MDA level, GSH-Px activity and SOD activity, were quantified via commercial detection kits following the standard protocols provided by the manufacturer [30].

### 2.8 Animal experiments and sample collection

Male C57BL/6J mice (6 weeks old, 18–22 g, SPF) and corn cob bedding were purchased from Changsheng Biotechnology Co., Ltd. (Liaoning, China). Under conditions of 21.0 ± 2 ℃ and 50–60% relative humidity. Each cage contained five mice, with free access to standard laboratory feed and drinking water throughout the experimental period. All experimental procedures were approved by Bioethics Committee of Chinese Academy of Agricultural Sciences (250808–04-GR). After adaptation period, mice were assigned to six groups (n = 10 per group): Control, DSS (Model), low-dose ASP (ASP-L), medium-dose ASP (ASP-M), high-dose ASP (ASP-H), and positive control (5-ASA) (Fig. S1). During 0–14 d period, Control group and Model group were given daily gavage of physiological saline, while ASP-L, ASP-M, and ASP-H groups were given daily gavage of 50, 150, and 450 mg/kg body weight (BW) of ASP, respectively (calculated based on the recommended human intake) [31], [32], [33]. 5-ASA group was orally administered 50 mg/kg BW of 5-ASA per day. During 0–7 d period, all mice were given sterile water [34]. During period of 8–14 days, Control group of mice drank sterile water, while other five groups of mice drank water containing 5% DSS to induce ulcerative colitis. Monitor and evaluate experimental mice daily. Following a 12 h overnight fast on experimental day 15, the orbital blood samples were harvested for subsequent detection. Let blood sample coagulate for 1 h and centrifuge at 3000 × g for 10 min to collect serum. Remove spleen and colon, and fix them with 4% paraformaldehyde for preservation. Rinse colon contents with cold physiological saline. Collect cecal contents and store them at −80 ℃ for subsequent analysis, spleen index is defined as ratio of spleen to body weight, measured in mg/g [35].

### 2.9 Disease activity index

Disease activity index (DAI), which integrates the scores for body weight reduction, fecal properties and bloody stool, was assessed for each mouse [36]. As a well‑established indicator for evaluating colitis severity in murine models, its concrete scoring rules are presented in Table S1.

### 2.10 ELISA

Cell supernatants and Serum levels of inflammatory cytokines (tumor necrosis factor-α (TNF-α), IL-17, IL-6, IL-4, IL-10 and TGF-β1) were quantified using ELISA kits, following the manufacturer’s instructions (Nanjing Jiancheng Company, Jiangsu, China).

### 2.11 Histopathologic evaluation

During sample collection, isolated colon tissues were immobilized in 4% paraformaldehyde. Tissue samples were sequentially dehydrated and paraffin-embedded to prepare for subsequent histological staining. Routine H&E staining was conducted on prepared slides, and histological morphological changes were observed and photographed at 100× and 400× microscopic magnifications [37].

### 2.12 Immunohistochemistry

Antigen retrieval of colon tissue slices was performed using citrate buffer solution. The processed sections were then probed with primary antibodies targeting ZO-1, Occludin and Claudin-1, followed by incubation with secondary antibodies. DAB chromogenic reagent was applied to visualize the positive staining regions. After being sealed with neutral gum, the sections were photographed via optical microscopy. Three randomly selected visual fields per sample were analyzed to quantify the area of positive protein expression [38].

### 2.13 Quantification of short‑chain fatty acids

Colon tissue samples were homogenized in a pre-cooled buffer containing 0.5% phosphoric acid. The homogenization process was performed at a frequency of 50 Hz for 3 min, followed by ultrasonic treatment for 10 min. After homogenization and sonication, the samples were centrifuged at 13000×*g* for 15 min at 4 °C. The resulting supernatant was collected, and N-butanol solvent supplemented with 10 μg/mL 2-ethylbutyric acid as the internal standard was added for metabolite extraction. Quantitative analysis of short-chain fatty acids (SCFAs) was performed using a gas chromatography-mass spectrometry (GC–MS) system. SCFA separation was conducted on an HP-FFAP capillary column (30 m × 0.25 mm × 0.25 μm) with a constant carrier gas flow rate of 1 mL/min. The column oven temperature program was optimized as follows: the initial temperature was set at 80 °C, increased to 120 °C at a heating rate of 40 °C/min, further elevated to 200 °C at 10 °C/min, and finally maintained at 230 °C for 3 min. The temperature of the ion source and quadrupole was stably maintained at 230 °C and 150 °C, respectively. Qualitative identification and quantitative analysis of SCFAs were completed using MassHunter software (v10.0, Agilent, USA). The software automatically matched characteristic ion fragments of target metabolites for structural verification. The final SCFA concentrations were calculated by substituting the sample peak areas into the established standard linear regression equations [39].

### 2.14 Analysis of gut microbiota

Mice fecal DNA was extracted following the protocol of the MagBeads FastDNA Kit for Soil (116564384) (MP Biomedicals, CA, USA). The V3–V4 variable region of the bacterial 16S rRNA gene was amplified via PCR using the specific primer pairs 341F (5′-CCTACGGGGCWGCAG-3′) and 806R (5′-GATACHVGGGTATCTAAT-3′). Amplified gene fragments were utilized for library construction with the Illumina TruSeq Nano DNA LT Library Prep Kit. Subsequently, paired-end sequencing (PE250) was carried out on the Illumina NovaSeq platform. Raw sequencing data were firstly demultiplexed using the Demux plugin. Primer sequences were trimmed with Cutadapt, and the DADA2 algorithm was applied for subsequent sequence quality filtering, denoising, sequence assembly, and chimera removal to obtain qualified clean sequences. Generate material property sequences (ASVs) and abundance information by combining sequences with 100% sequence similarity.

The R software package (v3.2.0) and QIIME2 2022.11 were the main tools used for sequence data analysis. The microbial community structure and abundance of each sample at different taxonomic ranks were determined using the QIIME2-derived ASV feature table. At the ASV level, alpha diversity indices were computed. Differentially abundant taxonomic entities between groups were identified using the linear discriminant analysis of effect size (LEfSe) approach. PICRUSt2 was used in combination with the MetaCyc (https://metacyc.org/) and KEGG (https://www.kegg. jp/) databases to undertake microbial functional prediction [40].

### 2.15 RNA-seq

RNA-seq was performed on colon tissues from randomly selected mice (n = 6 per group) in the Control, Model, and ASP-H groups using the Kidio Signal Cloud platform. Determine purity, concentration, and completeness of total RNA. Amplify concise cDNA and construct a cDNA library. Differentially expressed genes (DEGs) were identified using the DESeq2 R‑package, with heritability‑related genes filtered out from the analytical dataset. Cluster analysis is used to evaluate enrichment of DEGs in KEGG research methods, and corresponding volcano and heat maps are drawn. Relational summary indicates that if DEGs are enriched in studying environmental inheritance factor groups [41].

### 2.16 qRT-PCR

Total RNA was isolated from colon tissues using the TaKaRa MiniBEST Universal RNA Extraction Kit in accordance with the manufacturer’s standard protocols. Purified RNA was then reverse-transcribed into complementary DNA (cDNA) utilizing the PrimeScriptTM RT Reagent Kit with gDNA Eraser (TaKaRa). Perform fluorescence quantification to evaluate expression of genes (Roche diagnosis). Table S2 lists primer sequences for genes.

### 2.17 Statistical analyses

Values were depicted as the mean ± standard deviation. One-way analysis of variance (ANOVA) was conducted utilizing SPSS Statistics 27 (IBM, USA), with subsequent Duncan’s multiple range test. Data visualization was conducted using OriginLab 2025 (Northampton, Massachusetts, USA). Distinct lowercase letters denote statistically significant variations when *P* < 0.05.

## 3 Results

### 3.1 Effect of ultrasonic treatment on phenolic compounds in adlay seed

UPLC-QTOF-MS was used to qualitatively analyze chemical constituents of adlay seed extract (Table 1). A total of 14 compounds were identified. Based on structural characteristics, 5 phenolic acids (*p*-Coumaric acid, 3,4-Dihydroxybenzoic acid, *p*-Hydroxybenzoic acid, Caffeic acid, and Ferulic acid), 7 flavonoids (Epigallocatechin gallate, (−)-Epicatechin gallate, Rutin, Quercetin, Kaempferol, Naringenin, and Nobiletin), and 2 other polyphenols (Coniferyl alcohol and Syringaldehyde) were identified.

**Table 1:** Identification by UPLC-QTOF-MS of bioactive compounds in adlay seeds.

NO.	RT (min)	Chemical formula	MW	Exact mass (*m*/*z*)	MS (*m*/*z*) [M−H]^−^	MS^2^ (*m*/*z*)	Error (ppm)	Compounds
Phenolic acids								
1	0.61	C_7_H_6_O_4_	154	154.0266	153.0193	108.0220/109.0296	0.2274	3,4-Dihydroxybenzoic acid
2	2.60	C_7_H_6_O_3_	138	138.0317	137.0244	65.0393/93.0332	0.0445	*p*-Hydroxybenzoic acid
3	3.19	C_9_H_8_O_4_	180	181.0492	180.0372	107.0503/135.0452	−0.2808	Caffeic acid
4	5.84	C_9_H_8_O_3_	164	164.0473	163.0401	93.0342/119.0526	0.2637	*p*-Coumaric acid
5	12.43	C_10_H_10_O_4_	194	194.0579	193.0506	134.0375/149.0609/178.0271	0.1276	Ferulic acid

Flavonoids								
6	1.67	C_22_H_18_O_11_	458	458.0849	457.0776	125.0248/169.0142/305.0663	0.8545	Epigallocatechin gallate
7	3.85	C_22_H_18_O_10_	442	442.0899	441.0827	125.0213/169.0142/289.0717	0.1921	(−)-Epicatechin gallate
8	3.42	C_27_H_30_O_16_	610	610.1534	609.1461	271.0233/300.0125/301.0342	−0.2332	Rutin
9	13.06	C_15_H_10_O_7_	302	302.0426	301.0354	121.0299/151.0039/179.0344	−0.1878	Quercetin
10	14.81	C_15_H_10_O_6_	286	286.0477	285.0405	179.0343/257.0435	−0.2682	Kaempferol
11	13.12	C_15_H_12_O_5_	272	272.0685	271.0612	119.0508/151.0193	0.1449	Naringenin
12	17.36	C_21_H_22_O_8_	402	402.1233	401.9249	343.0822/358.1055/373.1281	0.4364	Nobiletin

Other polyphenols								
13	4.37	C_10_H_12_O_3_	180	180.0786	179.0714	119.0532/161.0614	−0.1162	Coniferyl alcohol
14	12.36	C_9_H_10_O_4_	182	183.0721	182.0349	123.0092/138.0331/166.0278	0.0407	Syringaldehyde

Using UHPLC-DAD architecture, corresponding standard samples were used as controls for quantitative analysis of compounds (Fig. 1). The content of identified compounds is shown in Table 2. For individual polyphenolic components, ultrasonic treatment markedly elevated the yield of total polyphenols at all tested power levels when compared with the untreated control group (*P* < 0.05), with an overall increase of 17.65% − 61.01%. As ultrasonic power increased polyphenol content gradually rose, however when power exceeded 600 W, the content began to decline. Among these, kaempferol, ferulic acid, and coniferyl alcohol were the phenolic compounds that showed the greatest increase in ultrasonically treated ASP. Under 300 W conditions, their contents increased by 92.59%, 80.01%, and 81.84%, respectively. At the same time, ferulic acid, and syringaldehyde were the major polyphenolic components in ASP. Compared with the untreated group, the contents of these three phenolic compounds increased by 10.51% − 52.48%, 22.25% − 80.01%, and 16.24% − 63.54%, respectively, after ultrasonic treatment. Under U300 W conditions, the absolute contents of naringenin, ferulic acid, and syringaldehyde reached 119.59 ± 10.31 mg/100 g, 68.69 ± 4.21 mg/100 g, and 66.15 ± 4.14 mg/100 g, but when ultrasonic power was too high, the contents of various polyphenolic compounds in ASP decreased.

**Fig. 1:**
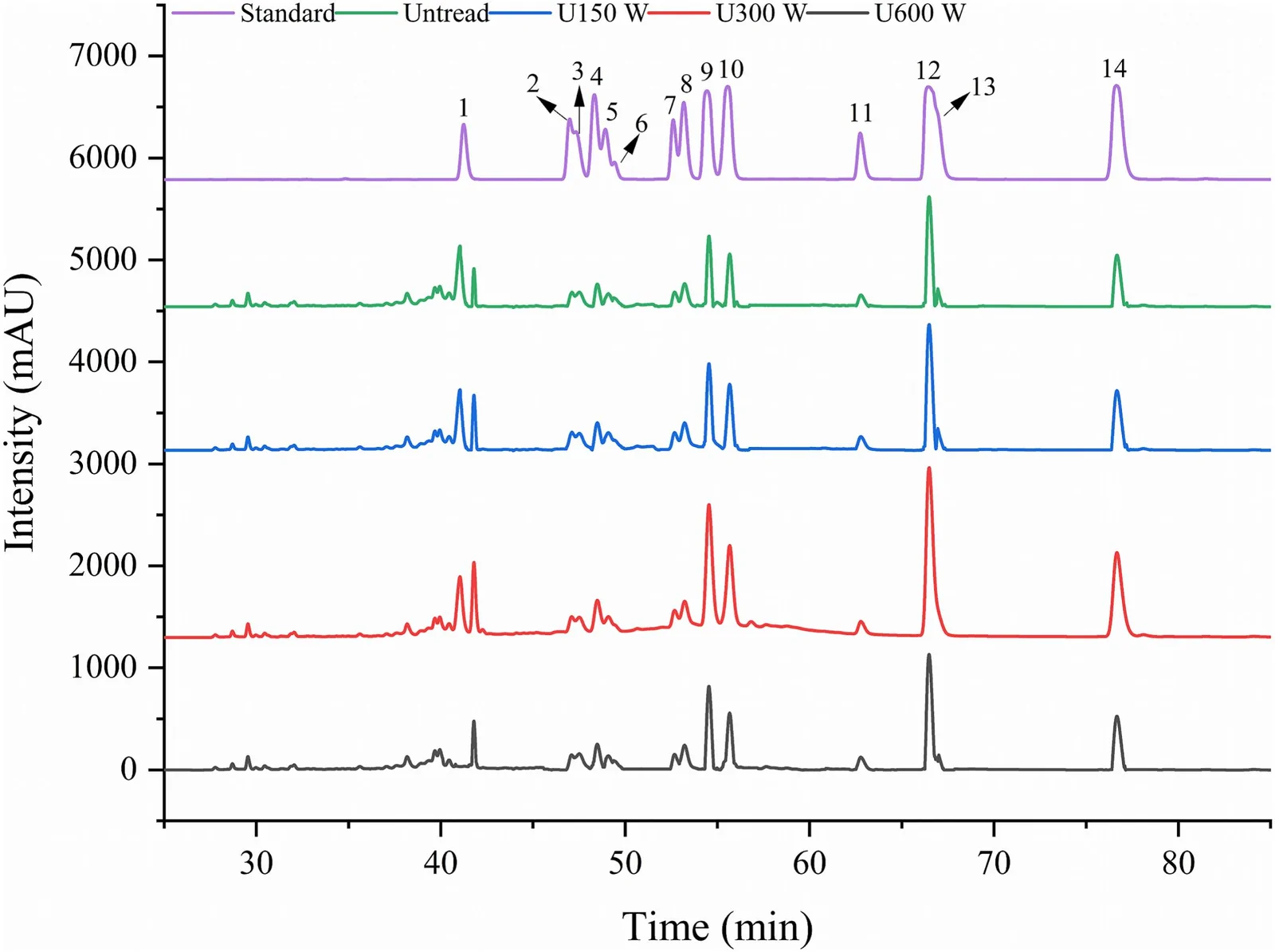
HPLC chromatogram (UV 280 nm) of phenolics from adlay seeds. Note: 1, 3,4-Dihydroxybenzoic acid (PA); 2, Epigallocatechin gallate (EGCG); 3, p-Hydroxybenzoic acid (PHA); 4, Caffeic acid (CA); 5, Rutin (RT); 6, (−)-Epicatechin gallate (ECG); 7, Coniferyl alcohol (CFA); 8, p-Coumaric acid (PCA); 9, Syringaldehyde (SA); 10, Ferulic acid (FA); 11, Quercetin (QE); 12, Naringenin (NG); 13, Kaempferol (KP); 14, Nobiletin (NT).

**Table 2:** Quantitative of the polyphenols from adlay seeds (mg/100 g).

Compounds	Untread	U150 W	U300 W	U600 W
3,4-Dihydroxybenzoic acid	18.14 ± 1.02^c^	25.47 ± 2.05^b^	30.68 ± 1.66^a^	26.65 ± 0.66^b^
Epigallocatechin gallate	5.23 ± 0.24^b^	7.99 ± 1.37^a^	8.63 ± 0.51^a^	7.83 ± 0.31^a^
*p*-Hydroxybenzoic acid	10.71 ± 1.13^b^	11.46 ± 0.35^b^	14.51 ± 1.41^a^	13.33 ± 0.48^a^
Caffeic acid	24.86 ± 1.64^c^	29.87 ± 2.26^b^	38.51 ± 1.47^a^	32.71 ± 1.40^b^
Rutin	10.48 ± 0.28^c^	14.32 ± 0.44^b^	18.32 ± 1.25^a^	17.14 ± 0.41^a^
(−)-Epicatechin gallate	12.11 ± 2.55^a^	11.59 ± 0.75^a^	14.11 ± 0.77^a^	12.28 ± 0.52^a^
Coniferyl alcohol	12.94 ± 0.98^c^	14.65 ± 0.83^c^	23.53 ± 1.62^a^	20.02 ± 1.70^b^
*p*-Coumaric acid	24.96 ± 1.45^d^	28.84 ± 1.44^c^	39.71 ± 2.60^a^	35.46 ± 1.27^b^
Syringaldehyde	40.45 ± 2.34^d^	47.14 ± 0.94^c^	66.15 ± 4.14^a^	56.94 ± 4.69^b^
Ferulic acid	38.16 ± 2.58^d^	46.65 ± 2.28^c^	68.69 ± 4.21^a^	57.74 ± 2.99^b^
Quercetin	7.01 ± 1.20^b^	9.02 ± 1.79^ab^	10.19 ± 0.33^a^	9.37 ± 0.31^a^
Naringenin	78.43 ± 3.19^c^	86.67 ± 3.56^c^	119.59 ± 10.31^a^	104.58 ± 7.47^b^
Kaempferol	10.12 ± 1.06^d^	13.13 ± 0.89^c^	19.49 ± 1.33^a^	16.50 ± 1.19^b^
Nobiletin	24.17 ± 1.59^c^	27.05 ± 1.18^c^	39.51 ± 3.04^a^	32.40 ± 2.43^b^
Total	317.76 ± 21.18^d^	373.86 ± 20.08^c^	511.64 ± 34.62^a^	442.95 ± 25.55^b^

Different lowercase letters (a-d) indicate significant differences between groups (*P* < 0.05).

### 3.2 Effect of ultrasonic treatment on the anti-inflammatory activity of adlay seed polyphenol extracts

The anti-inflammatory activity of ASP is shown in Fig. 2. LPS stimulation significantly induced cellular inflammatory damage. Compared with the Control group, the Model group exhibited significantly reduced cell viability (*P* < 0.05), and markedly elevated levels of intracellular LDH, ROS (Fig. 2A-C), MDA (Fig. 2F), and in the supernatant (127.09%, 115.67%, and 626.72%), while the activity of the antioxidant enzymes SOD and GSH-Px (Fig. 2D, E) decreased substantially (55.76% and 68.83%). Meanwhile, the secretion levels of pro-inflammatory cytokines (TNF-α, IL-6, and IL-17) were markedly increased by 315.44%, 209.98%, and 122.82%, respectively (Fig. 2G–I). In contrast, the production of anti-inflammatory mediators, including IL-4, IL-10, and TGF-β1, was prominently decreased by 65.94%, 66.41%, and 53.52% (Fig. 2J–L). These findings indicated that LPS stimulation successfully induced in vitro inflammatory injury, which was manifested by disrupted oxidative stress homeostasis, cell membrane impairment, and an imbalance between pro‑inflammatory and anti‑inflammatory cytokine release.

**Fig. 2:**
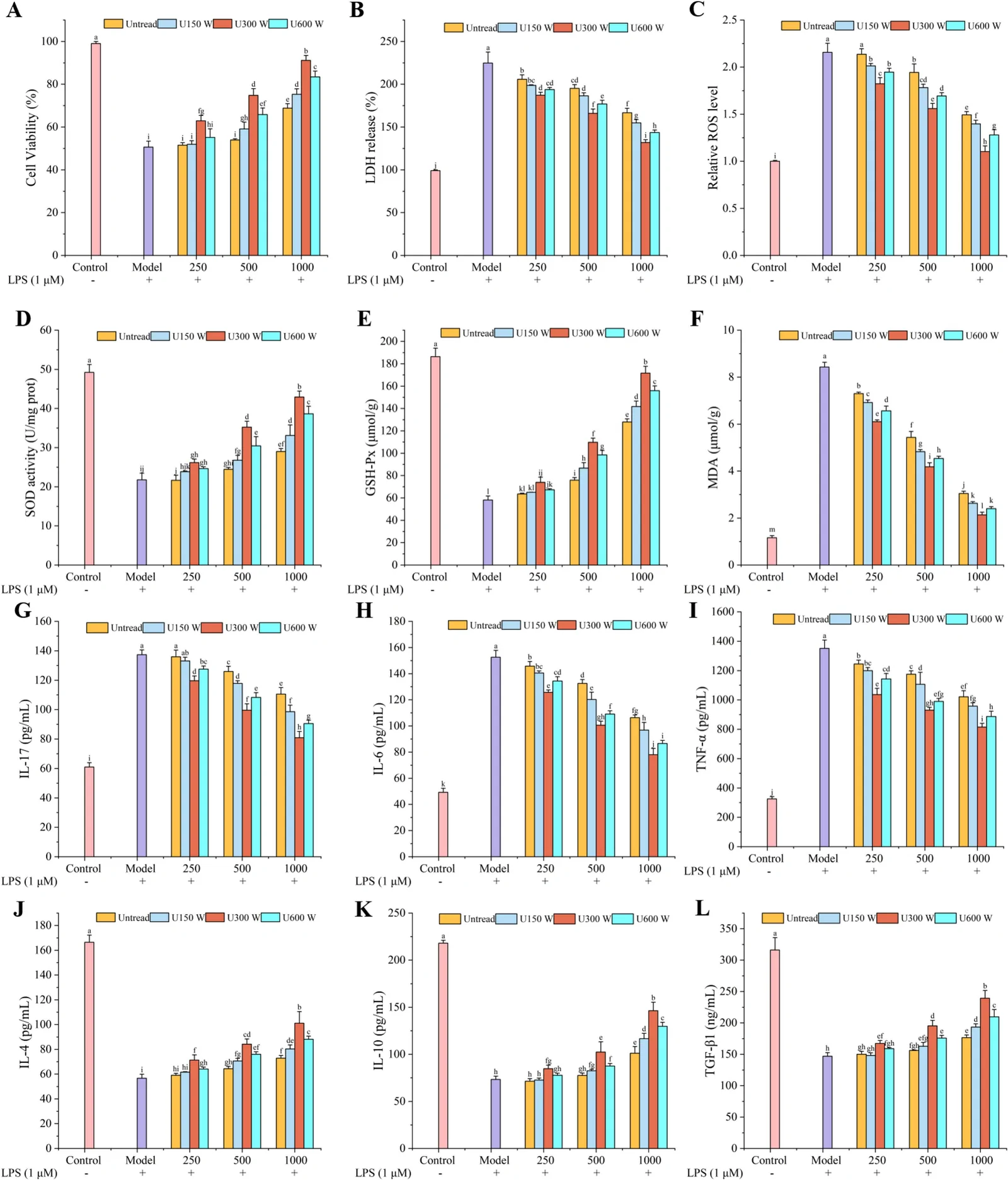
Anti-inflammatory of adlay seed polyphenols on LPS-induced inflammation. (A) Cell viability. (B) LDH release. (C) ROS level. (D) SOD activity. (E) GSH-Px activity. (F) MDA. (G) IL-17. (H) IL-6. (I) TNF-α. (J) IL-4. (K) IL-10. (L)TGF-β1. Different lowercase letters (a-m) indicate significant differences between groups (*P* < 0.05).

Compared with the Control group, ASP treatment reduced cellular oxidative stress levels in a dose-dependent manner. At the same dose, ultrasonically treated ASP exhibited superior anti-inflammatory activity compared to untreated ASP. Furthermore, as ultrasonic power increased, the anti-inflammatory activity of ASP significantly improved. However, when ultrasonic power reached 600 W, the anti-inflammatory activity of ASP slightly decreased. ASP treated with different ultrasonic intensities showed significantly lower levels of intracellular ROS, MDA, and LDH release in the supernatant compared to the Model group (*P* < 0.05). In the ASP-H group, these three biomarkers were reduced by 30.69% − 48.84%, 63.82% − 74.61%, and 25.83% − 41.29%, respectively. Compared with the untreated ASP group at the same dose, U300 W ASP further reduced ROS, MDA, and LDH by 26.17%, 29.93%, and 20.86%, respectively. At the same time, the levels of the SOD and GSH-Px were significantly enhanced by 33.19% − 97.15% and 120.29% − 195.49%, respectively. Compared with untreated ASP, the U300 W showed increases in the activities of these two antioxidant enzymes by 48.02% and 34.13%, respectively. These results indicate that ASP can scavenge excess reactive oxygen species, alleviate lipid peroxidation damage, maintain cell membrane integrity, and restore intracellular antioxidant defense systems. Furthermore, these capabilities can be further enhanced through appropriate ultrasonic pretreatment.

In terms of inflammatory responses, ASP intervention exerted significant dose-dependent inhibitory effects on the secretion of pro-inflammatory mediators, including TNF-α, IL-6, and IL-17. In particular, the high-dose ASP combined with 300 W ultrasonic treatment decreased the levels of these three pro-inflammatory cytokines by 20.19%, 26.57%, and 26.85%, respectively, relative to the untreated model group. Meanwhile, the release of anti-inflammatory factors (IL-4, IL-10, and TGF-β1) was markedly upregulated (*P* < 0.05), with elevation ranges of 28.54%–78.44%, 38.14%–99.74%, and 20.16%–62.84% compared with the Model group. Under identical dosage conditions, ultrasonically processed ASP presented superior anti-inflammatory capacity compared with non-ultrasonic ASP samples. Notably, ASP treated under the optimal ultrasonic power of 300 W exhibited the most pronounced effects, with stronger suppression of pro-inflammatory cytokine production and more effective promotion of anti-inflammatory factor secretion.

In summary, ASP achieves bidirectional regulation of oxidative stress and inflammatory states. It alleviates oxidative damage by reducing ROS, MDA, and LDH levels, and enhances cellular antioxidant capacity by increasing SOD and GSH-Px activity. Furthermore, ASP restores the pro-inflammatory/anti-inflammatory balance by downregulating TNF-α, IL-6, and IL-17 and upregulating IL-4, IL-10, and TGF-β1, thereby alleviating LPS-induced cellular inflammatory damage. Notably, these anti-inflammatory effects are regulated by two key factors: increasing the ASP dose generally enhances the anti-inflammatory effect, while appropriate ultrasonic treatment further enhances ASP’s anti-inflammatory capacity, indicating that ultrasonic-assisted extraction is an effective strategy for improving the bioactivity of ASP.

### 3.3 Effects of adlay seed polyphenol extracts on symptoms in colitis mice

A colitis model was further induced using 5% DSS, and ASP extracted using the U300 W was selected to evaluate its efficacy in alleviating colitis in mice and to investigate the underlying mechanism (Fig. 3). During the 1 week of ASP administration, there were no significant changes in body weight or DAI scores, indicating that ASP had no adverse effects on the mice. Starting from the 2 week of DSS exposure, the Model group began to lose body weight from day 10 onward. Compared with the Control group, body weight had decreased significantly by nearly 22% by day 14, and DAI scores were significantly elevated (*P* < 0.001) (Fig. 3A, B). Compared with the Model group, ASP administration significantly alleviated colitis-induced weight loss and significantly reduced DAI scores in a dose-dependent manner (*P* < 0.05). By day 14, they had increased by 1.54% − 5.64% and decreased by 11.68% − 40.02%, respectively. Furthermore, ASP effectively alleviated DSS-induced splenomegaly (Fig. 3C). On day 14, the spleen index was reduced by 5.87% compared to the Model group.

**Fig. 3:**
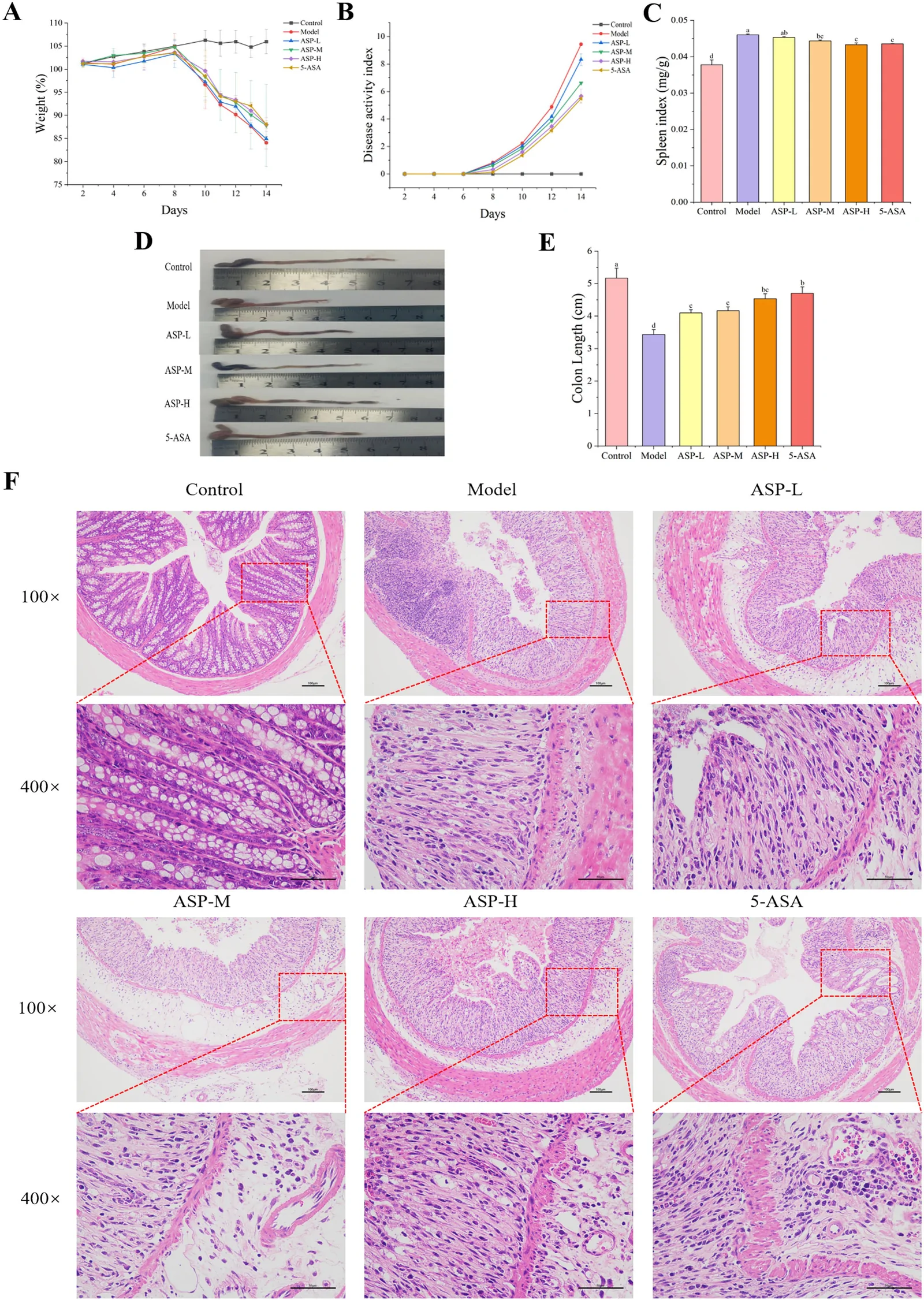
Effect of ASP trial on colitis induced by DSS. (A) Changes of body weight of mice in different treatment groups. (B) DAI score of mice. (C) Spleen index of mice. (D) Macroscopic map of mice colon. (E) Length changes of mice colon. (F) HE staining. Different lowercase letters (a-d) indicate significant differences between groups (*P* < 0.05).

Shortened colon length is one of the primary manifestations of intestinal damage (Fig. 3D, E). Colon length in the Model group decreased from 5.17 ± 0.31 cm in the Control group to 3.43 ± 0.15 cm. In the ASP intervention group, colon length ranged from 4.10 ± 0.10 to 4.53 ± 0.15 cm, indicating a significantly slower rate of colon shortening compared to the Model group. Notably, the therapeutic outcomes of the ASP‑H group were statistically indistinguishable from those of the 5‑ASA positive control, implying that high‑dose ASP could achieve similar colitis‑ameliorating efficacy as 5‑ASA intervention. Histopathological observation of colon tissues via H&E staining (Fig. 3F) showed severe intestinal morphological destruction in the Model group. Specifically, DSS stimulation caused extensive epithelial and goblet cell loss, distorted crypt architecture, and robust inflammatory infiltration in the colonic mucosa. In comparison, ASP treatment effectively alleviated intestinal histological lesions. The ASP-treated mice presented relatively complete tissue morphology with well-preserved goblet cells. Moreover, mucosal structural integrity was largely maintained, accompanied by reduced goblet cell depletion and mitigated inflammatory infiltration. Collectively, these histological findings confirmed that ASP could efficiently protect intestinal tissue against DSS-induced inflammatory injury.

### 3.4 Effects of adlay seed polyphenol extracts on the intestinal barrier and inflammatory factors in colitis mice

To further elucidate the regulatory effects of ASP on intestinal barrier dysfunction and inflammatory responses in DSS-induced colitis, the expression of colonic tight junction proteins and inflammatory cytokines was determined (Fig. 4). Immunohistochemical analysis revealed that the protein levels of ZO-1, Occludin, and Claudin-1 were markedly downregulated in the Model group compared with the Control group, with reduction rates of 71.00%, 57.28%, and 50.51%, respectively (Fig. 4A–D). In contrast, ASP treatment significantly upregulated the expression of these tight junction proteins in a dose-dependent manner. Specifically, high-dose ASP intervention increased the levels of ZO-1, Occludin, and Claudin-1 by 65.52%, 54.55%, and 61.22%, respectively, indicating that ASP could effectively ameliorate DSS-triggered intestinal barrier impairment.

**Fig. 4:**
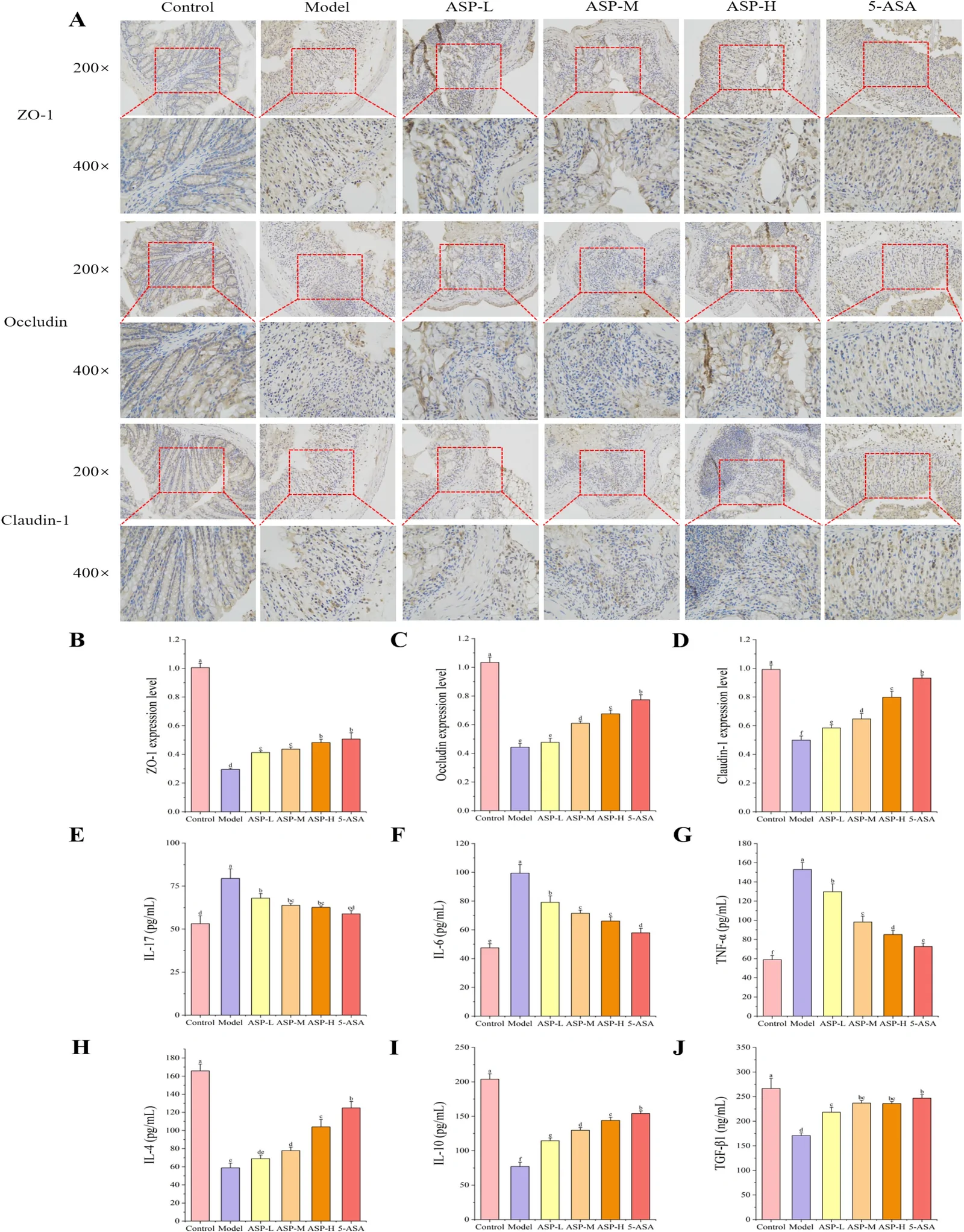
Effect of ASP on the intestinal barrier and inflammatory factors in mice with colitis. (A) Immunohistochemistry of tight junction proteins. (B-D) Colon tissues levels of tight junction proteins. (E-G) Levels of the pro-inflammatory cytokine IL-17, IL-6 and TNF-α. (H-J) Levels of the anti-inflammatory cytokine IL-4, IL-10 and TGF-β1. Different lowercase letters (a-f) indicate significant differences between groups (*P* < 0.05).

Excessive secretion of pro-inflammatory cytokines (TNF-α, IL-6, and IL-17) is an important driving factor for intestinal tissue damage during development of colitis. Consistent with barrier injury phenotype, serum levels of pro-inflammatory cytokines in model group significantly increased, with IL-17, IL-6, and TNF-α values increasing by 49.33%, 109.43%, and 159.71%, respectively. Compared to Control group (Fig. 4E-G), serum levels of anti-inflammatory mediators (IL-4, IL-10, and TGF-β1) in Model group decreased by 64.56%, 62.21%, and 35.97%, respectively (Fig. 4H-J). Confirming that DSS treatment caused severe systemic inflammatory imbalance. Compared with Model group, ASP treatment significantly reversed this inflammatory state (*P* < 0.05). Inhibited release of pro-inflammatory factors, and enhanced expression of anti-inflammatory cytokines. In ASP-H group, levels of IL-17, IL-6, and TNF − α decreased by 21.15%, 33.47%, and 44.31%, respectively. While levels of IL-4, IL-10, and TGF-β1 increased by 77.00%, 86.84%, and 38.13%, respectively. ASP restores integrity of intestinal barrier and suppresses excessive inflammatory response to alleviate DSS induced colitis.

### 3.5 Regulatory effects of adlay seed polyphenol extracts on the gut microbiota of colitis mice

To further characterize the regulatory role of ASP on intestinal microflora structure, high-throughput 16S rRNA sequencing was conducted to analyze gut microbiome alterations in colitis mice (Fig. 5). The Venn diagram (Fig. 5A) shows that the Control, Model, and ASP groups had 391, 317, and 217 OTUs, respectively. There were 186 shared OTUs across all groups. A total of 104 and 27 exclusive OTUs were detected in the Control and Model groups, respectively. The divergent OTU profiles demonstrated obvious discrepancies in gut microbial structure between normal and colitic mice, and specific microbial species were uniquely present following ASP treatment. The abundance of the microbiome diversity in colitis mice was analyzed using α diversity metrics. The α-diversity indices Shannon and Simpson can be used to reflect community diversity, the higher the Shannon value and the lower the Simpson value, the higher the community diversity. DSS-induced colitis significantly altered microbial diversity indices. Specifically, the Model group displayed a 14.61% decrease in the Shannon value and a 175.00% elevation in the Simpson value relative to the Control group (*P* < 0.05) (Fig. 5B). Compared with the Model group, the ASP group showed a significantly higher Shannon value (5.50%) and a significantly lower Simpson value (54.55%) (*P* < 0.05) (Fig. 5C). This indicates that modeling reduced the community diversity of the gut microbiota in mice, while ASP intervention mitigated the extent of community diversity disruption in the gut microbiota of mice with colitis. At the same time, ASP alleviated the damage to the abundance of the gut microbiota, bringing it closer to that of the normal group.

**Fig. 5:**
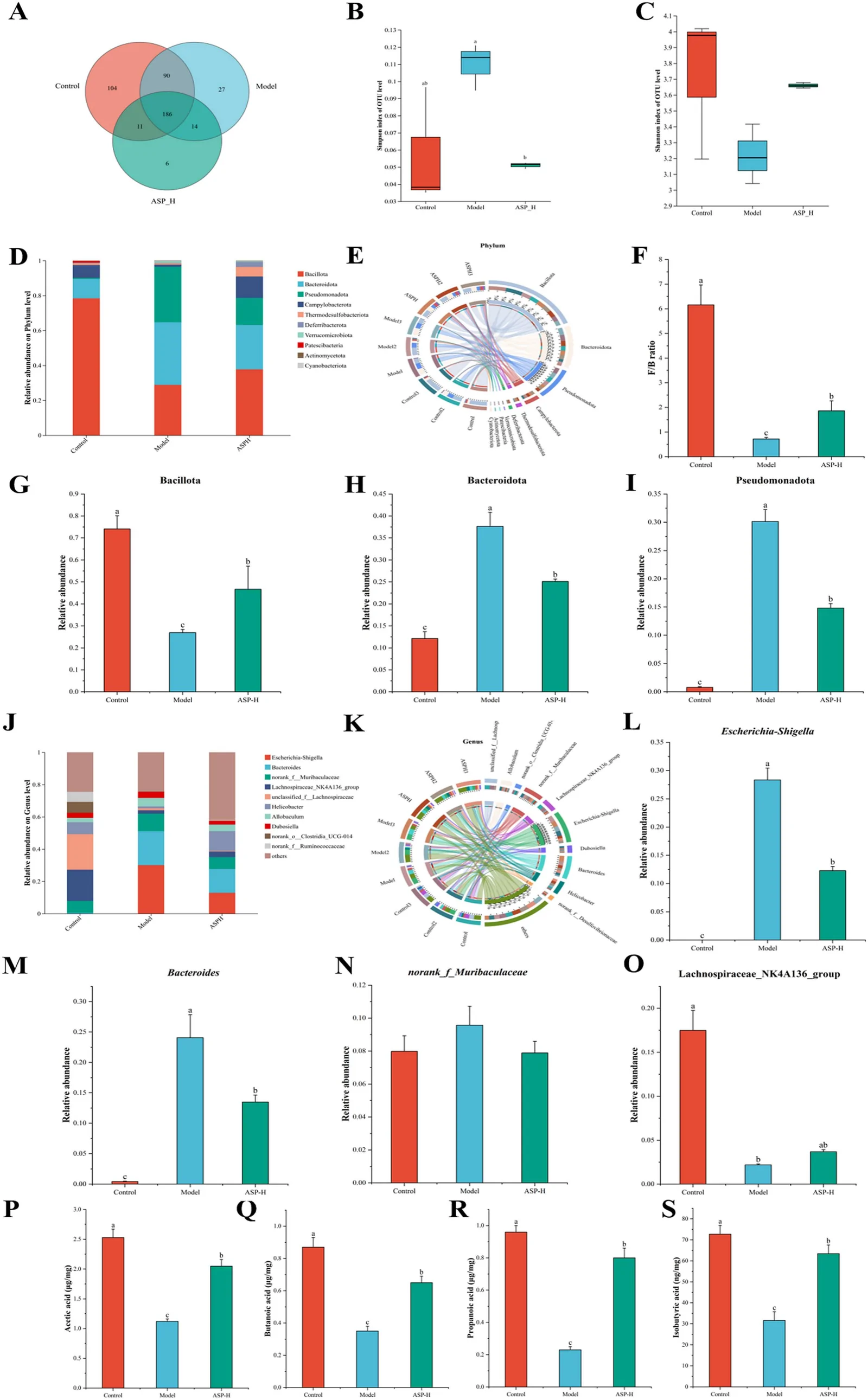
Effect of ASP on gut microbial composition. (A) Venn diagram. (B) Simpson index. (C) Shannon index. Figure D-I depict the relative abundance of different phylum of gut microbiota. (D) Overall microbiota. (E) Circos plot. (F) F/B ratio (Bacillota (formerly Firmicutes)/ Bacteroidota (formerly Bacteroidetes)). (G)Bacillota. (H) Bacteroidota. (I) Pseudomonadota. Figure J-O depict the relative abundance of different genus of gut microbiota. (J) Overall microbiota. (K) Circos plot. (L) *Escherichia-Shigella*. (M) *Bacteroides*. (N) *norank-f-Muribaculaceae*. (O) *Lachnospiraceae-NK4A136-group*. (P) Changes in acetic acid level. (Q) Changes in butanoic acid level. (R) Changes in propanoic acid level. (S) Changes in isobutyric acid level. Different lowercase letters (a-c) indicate significant differences between groups (*P* < 0.05).

The fecal microbial composition at the phylum level is presented in Fig. 5D–I. Relative to the Control group, the Model group showed a 60.81% significant decrease in the relative abundance of Bacillota (formerly Firmicutes), whereas the abundances of Bacteroidota (formerly Bacteroidetes) and Pseudomonadota (formerly Proteobacteria) were markedly elevated by 216.67% and 36.50-fold, respectively. In comparison with the Model group, ASP treatment effectively reversed the dysregulated microbial composition at the phylum level: ASP significantly upregulated the relative abundance of Bacillota by 80.77% and reduced the abundances of Bacteroidota and Pseudomonadota by 34.21% and 50.67%, respectively. The Bacillota (formerly Firmicutes)/Bacteroidota (F/B) ratio, a critical biomarker reflecting gut microbial homeostasis, was remarkably decreased in DSS-challenged mice, while ASP intervention significantly restored the F/B ratio by 158.33%. Genus-level microbial analysis further revealed distinct microbial alterations among groups (Fig. 5J–O). The Model group exhibited significant enrichment of pro-inflammatory genera *Escherichia-Shigella* and *Bacteroides* (*P* < 0.001), along with a pronounced reduction in the beneficial *Lachnospiraceae_NK4A136_group* (*P* < 0.05). Compared with the Model group, ASP treatment dramatically suppressed the relative abundances of *Escherichia-Shigella*, *Bacteroides*, and *norank_f__Muribaculaceae* by 57.14%, 45.83%, and 12.63%, respectively. Meanwhile, the abundance of *Lachnospiraceae_NK4A136_group* was increased by 68.49% after ASP supplementation. These results demonstrated that ASP could effectively reshape the overall structure of gut microbiota in colitic mice.

Short-chain fatty acids (SCFAs), pivotal microbial metabolites that maintain intestinal homeostasis, were further quantified in mouse cecal contents. The results indicated that the concentrations of acetic acid, butyric acid, propionic acid, and isobutyric acid were prominently depleted in the Model group. In contrast, ASP treatment significantly restored SCFA accumulation, with the levels of acetic acid, butyric acid, propionic acid, and isobutyric acid increased by 83.04%, 247.83%, 83.33%, and 100.82%, respectively (*P* < 0.001) (Fig. 5P–S). Collectively, these findings suggested that ASP alleviated DSS-induced colitis via remodeling gut microbial community structure and elevating microbial-derived SCFA production.

Based on LDA scores, LefSe further identified marker species specific to the Control, Model, and ASP groups (Fig. 6A). *c_Actinobacteria*, *g_Corynebacterium*, and *g_Mammaliicoccus* were found to be specific to the Model group. In the ASP group, *g_Alistipes*, *g_norank_f_Anaerovoracaceae*, and *g_norank_f_Erysipelotrichaceae* underwent changes and became dominant genera. Consistent with the genus-level microbial profiling results, PICRUSt2-based functional prediction was subsequently performed to elucidate gut microbial functional variations across the Control, Model and ASP intervention groups (Fig. 6B). The results indicated that the predicted KEGG metabolic pathways primarily involved lipid metabolism disorders, abnormal cell motility, and inflammation-related signaling pathways. Collectively, ASP restored gut microbiota homeostasis via suppressing the enrichment of pathogenic bacteria, including *Escherichia-Shigella*, *Bacteroides* and *norank_f_Muribaculaceae*, which contributed to the alleviation of DSS-induced acute colitis. Its mechanism of action may involve suppressing overactivated inflammatory pathways and reshaping host metabolic functions, thereby alleviating DSS-induced functional abnormalities.

**Fig. 6:**
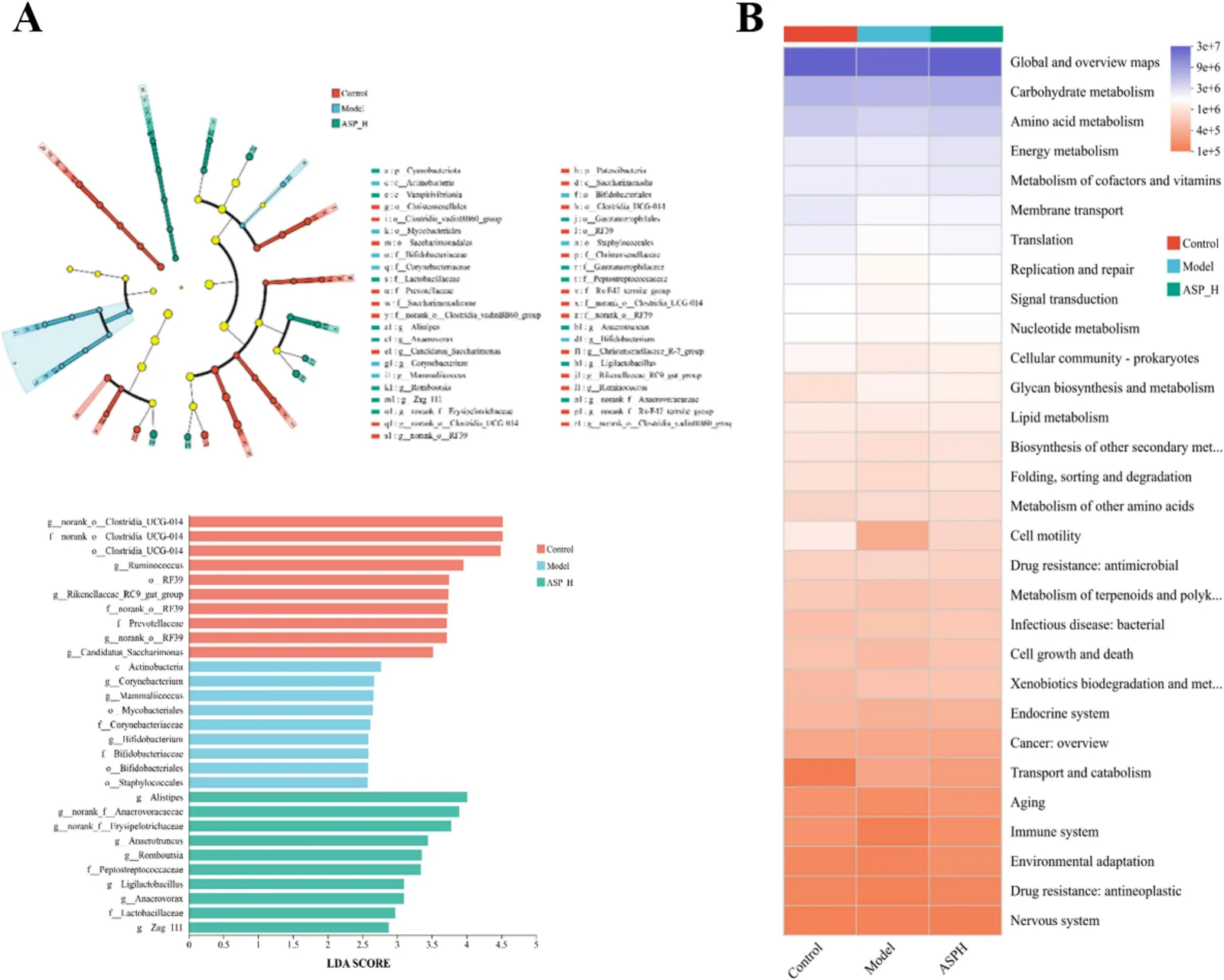
LEfSe and PICRUSt2 analysis of gut microbiome. (A) LEfSe analysis. (B) PICRUSt2 functional prediction.

### 3.6 Adlay seed polyphenol extracts alleviates colitis signaling pathways

To further elucidate the therapeutic mechanisms of ASP in colitis, RNA-seq analysis was conducted (Fig. 7). ASP treatment affected a total of 173 genes in the intestines of colitis-affected mice (Fig. 7A). Compared with the Control group, the Model group significantly increased the expression of 113 genes in mouse colon tissue while decreasing the expression of 166 genes (Fig. 7B). Compared with the Model group, ASP intervention led to a significant increase in the expression of 305 genes while decreasing the expression of 354 genes (Fig. 7C). Based on the 173 screened common target genes, GO functional annotation and KEGG pathway enrichment analyses were performed via the Metascape database to systematically explore the potential molecular mechanisms by which ASP alleviates colitis. GO analysis (Fig. 7D) indicated that differentially expressed genes (DEGs) were significantly enriched in various molecular functions, such as binding, catalytic activity, and catalytic activity. Additionally, these DEGs were associated with multiple cellular components, including intracellular and protein-containing complexes. These screened DEGs participated in a wide range of biological functional pathways, mainly covering cellular processes, regulatory biological behaviors, and metabolic processes. Furthermore, GO enrichment analysis of DEGs (Fig. 7E) revealed significant enrichment in various biological processes, including immune response, defense response to bacteria, and phagocytosis. Among these, the immune response is a key biological driver of colonic mucosal inflammation. The GO enrichment results suggest that ASP may alleviate symptoms in colitis model mice by regulating immune response, neutrophils, and intercellular signaling.

**Fig. 7:**
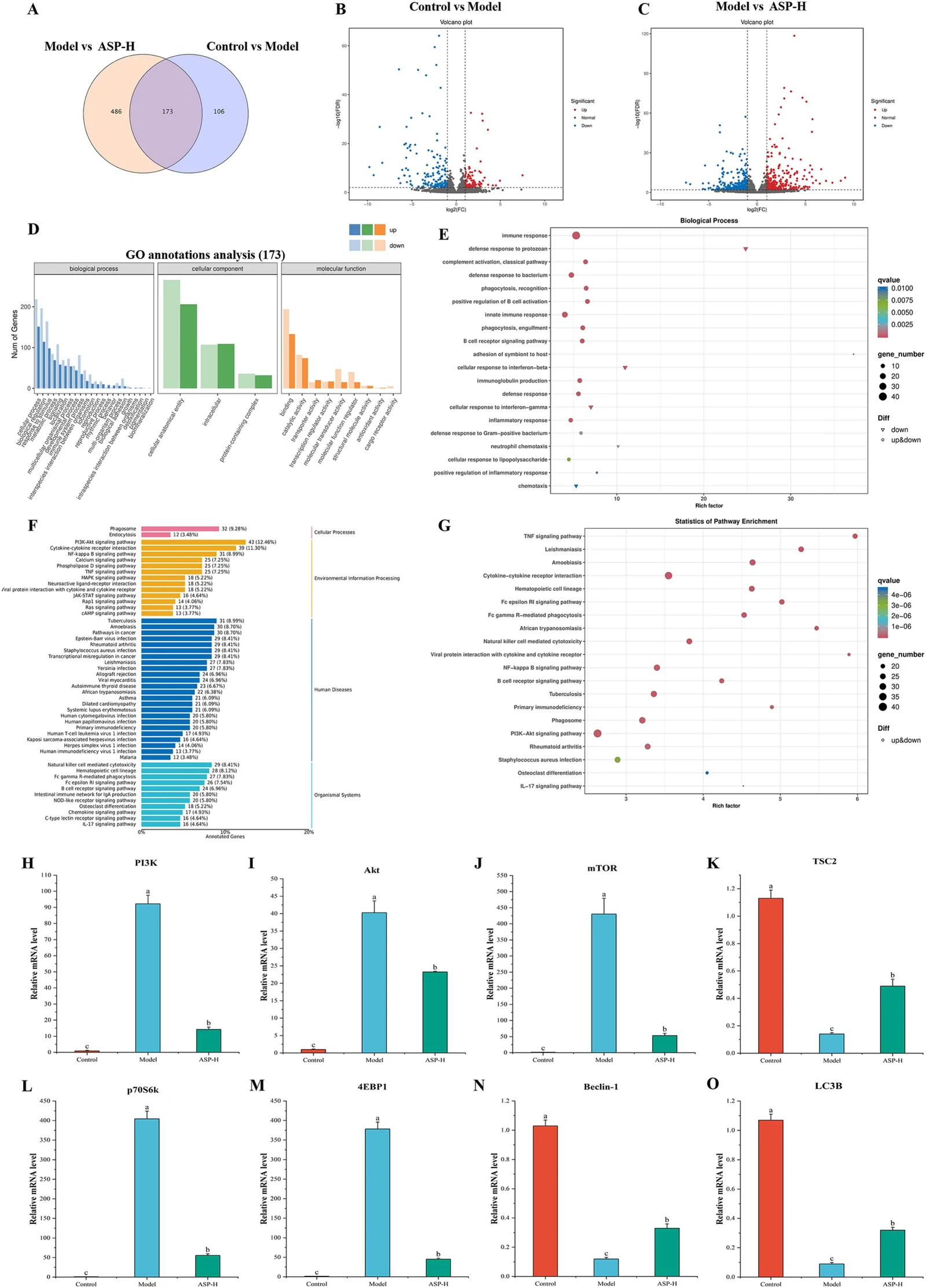
Transcriptomic analyses of the ability of ASP to alleviate DSS-induced colitis. (A) Venn diagram. (B) DEGs between Control and Model group. (C) DEGs between Model and ASP-H group. (D) GO function annotation analysis. (E) GO function enrichment analysis. (F) Functional annotation analysis of KEGG functions critically. (G) Functional enrichment analysis of KEGG functions critically. Figure H-O was determined by qRT-PCR. (H) PI3K mRNA expression. (I) Akt mRNA expression. (J) mTOR mRNA expression. (K) TSC2 mRNA expression. (L) p70S6k mRNA expression. (M) 4EBP1 mRNA expression. (N) Beclin-1 mRNA expression. (O) LC3B mRNA expression. Different lowercase letters (a-c) indicate significant differences between groups (*P* < 0.001).

KEGG pathway enrichment analysis of DEGs identified several significantly enriched signaling cascades, including the phagosome, endocytosis, and PI3K–Akt signaling pathways (Fig. 7F). Further functional pathway annotation demonstrated that the TNF signaling pathway, PI3K–Akt signaling pathway, and cytokine–cytokine receptor interaction were prominently enriched (Fig. 7G). Integrative analysis of RNA‑seq transcriptomic data consistently highlighted significant enrichment of the PI3K–Akt pathway, implying that ASP may ameliorate DSS-induced colitis by suppressing pro-inflammatory signaling cascades. Subsequent qRT-PCR validation was performed to verify this regulatory mechanism. As illustrated in Fig. 7H–O, the mRNA levels of PI3K, Akt, and mTOR were significantly upregulated in the Model group by 104.82%, 40.68%, and 367.79%, respectively (*P* < 0.05), indicating robust hyperactivation of the PI3K/Akt/mTOR pathway during colitis progression. In contrast, high-dose ASP treatment markedly inhibited the overactivation of this pathway. Compared with the Model group, the ASP-H group exhibited decreased mRNA expression of PI3K, Akt, and mTOR by 84.63%, 42.26%, and 87.68% (*P* < 0.05). Additionally, ASP intervention effectively reversed the abnormal expression of upstream (TSC2) and downstream (p70S6k, 4EBP1, Beclin-1, and LC3B) genes involved in the mTOR signaling axis. Collectively, these results demonstrated that adlay seed polyphenol extract could alleviate intestinal inflammatory injury by restraining aberrant activation of the core PI3K/Akt/mTOR inflammatory pathway.

## 4 Discussion

Adlay is highly nutritious and is hailed as the “King of the Grasses”. In the Shennong Bencao Jing, adlay seed is classified as a “superior-grade” herb and can be used as both a nutritional food and a medicinal preparation. Studies have shown that adlay seeds possess hypoglycemic activity, which is attributed to their high polyphenol content [13]. Recent evidence suggests that a diet rich in polyphenolic compounds can serve as an important adjunctive measure for reducing inflammation, particularly colitis [8]. However, people have limited understanding of composition of adlay seed polyphenol extracts (ASP), their therapeutic efficacy for colitis, and potential molecular mechanisms involved. This study identified phenolic components of ASP, significantly expanding field of chemical composition known in this edible crop and filling gap.

Through UHPLC-QTOF-MS/MS analysis, we identified 14 phenolic compounds in ASP. Many compounds have significant anti-inflammatory and intestinal protective effects. Ultrasonic processing resulted in maximum increase of kaempferol, ferulic acid, and coniferyl alcohol in ASP. Previously, resveratrol regulated TLR4 and oxidative stress signaling pathways to protect intestinal barrier [42]. Ferulic acid can alleviate ulcerative colitis in mice, which is associated with its capacity to remodel gut microbial composition and trigger the aryl hydrocarbon receptor (AhR)-dependent anti-inflammatory pathways [43]. Meanwhile, naringenin can suppress oxidative stress markers and inhibit inflammatory responses, thereby alleviating colonic damage [44]. Importantly, the analysis in this study indicates that these four polyphenols are present in relatively high abundances in ASP, confirming their significant contribution to the enhanced overall efficacy of ASP following ultrasonic treatment. Furthermore, polyphenols from various sources such as Fuzhuan tea, and green peas exhibit anti-colitis effects through mechanisms similar to those of ASP components, further highlighting the therapeutic potential of polyphenol-rich extracts [45], [46]. Nevertheless, because ASP is a complex crude extract, the biological effects observed in the cellular and animal experiments should be attributed to the ASP extract as a whole, and the possible contributions of other identified or unidentified co-extracted constituents cannot be excluded. However, researchers have found that the minor non-polyphenolic compounds in the extract exhibit significantly weaker antioxidant activity than the polyphenolic compounds, and the extract’s free radical scavenging capacity is primarily determined by its phenolic profile [47], [48]. At the same time, numerous studies have identified phenolic acids and flavonoids as the key bioactive constituents of adlay seeds, underlying their antioxidant, anti-inflammatory and other health-promoting effects [13], [15]. Therefore, although the extract as a whole account for the observed effects, polyphenols are very likely to be its predominant active constituents.

This provides corresponding evidence for anti-inflammatory activity of ASP, and maintenance of anti-inflammatory activity of UC indicates potential evaluation of sustained candidates in development of functional foods. Simultaneously, ASP extraction results at different ultrasonic power levels indicate that ultrasonic power can significantly regulate the polyphenol content and anti-inflammatory activity of ASP. Taken together, all detected indices rose first and subsequently declined, whereby 300  W represented the most favorable power condition for ultrasonic‑assisted extraction. Neither excessively low nor excessively high power levels can achieve both efficient polyphenol extraction and superior anti-inflammatory activity. Within the 150–300 W power range, as ultrasonic power increases, the cavitation and mechanical vibration effects gradually intensify, effectively disrupting the cell wall structure of adlay seeds and promoting the full release of intracellular polyphenolic bioactive compounds. This leads to a gradual increase in polyphenol extraction yield, while the inhibitory activity of the extracted polyphenols against cellular inflammation also continues to strengthen [19]. However, when the ultrasonic power is increased to 600 W, the excessively high ultrasonic energy triggers excessive cavitation and thermal effects. This not only causes structural damage and oxidative degradation of the active polyphenolic components − leading to a decrease in the effective polyphenol content − but also reduces the biostability of the active components, ultimately resulting in a significant decline in anti-inflammatory activity [49]. Previous studies have verified that ultrasonic power serves as a critical determinant for both extraction yield and functional activity of phytochemical polyphenols in ultrasonic-assisted extraction systems. Moderate ultrasound treatment can maximize the release of active components, whereas excessive ultrasound treatment can damage the active hydroxyl structures of polyphenols and weaken their biological functions [21].

Subsequently, the ultrasonic power group (U300 W) exhibiting the highest ASP activity was selected, and a validation study of ASP’s biological activity was conducted using a well-established DSS-induced colitis model in mice [34]. The core phenotypic indicators used to evaluate the polyphenol’s ability to alleviate DSS-induced colitis were body weight, DAI score, and colon HE staining [36]. DSS disrupts the intestinal epithelial barrier and induces local inflammation, resulting in persistent weight loss and significantly elevated DAI scores in the model mice. Histopathological sections revealed crypt disorganization, goblet cell depletion, extensive inflammatory infiltration, and mucosal ulcers. Polyphenol intervention dose-dependently alleviated weight loss in mice, reduced DAI scores, and improved symptoms of diarrhea and bleeding. HE staining confirmed that polyphenols restored crypt structure, replenished goblet cells, reduced inflammatory infiltration, and repaired colonic mucosal damage. Studies have shown that the inflammatory response is a central pathological feature of colitis; persistent inflammation triggered by intestinal mucosal immune dysregulation is the primary cause of intestinal mucosal damage, ulcer formation, and disease progression [50]. As pivotal pro‑inflammatory mediators, IL‑17, IL‑6 and TNF‑α dominate the onset, amplification and sustained progression of colitis‑associated inflammation. Over‑secretion of these cytokines worsens mucosal lesions and drives disease deterioration [44]. Conversely, anti‑inflammatory cytokines exert core functions in dampening and terminating host inflammatory responses, preserving immune balance, and restoring impaired intestinal tissues. Their insufficient expression can lead to sustained spread of inflammatory response and hinder tissue repair [8]. Among these, IL-4 is a context-dependent cytokine. IL-4/IL-13 signaling mediated by the IL-4Rα receptor drives the differentiation of tissue-repairing (M2a) macrophages, and gene knockout of this signaling pathway significantly impedes the recovery from DSS-induced colonic injury [51]. However, in the inflamed colon of UC patients, IL-4 expression is significantly reduced; its restoration promotes IL-4/STAT6-mediated M2 macrophage polarization, accompanied by increased levels of IL-10 and TGF-β and suppression of TNF-α [52]. In this study, IL-4 was significantly suppressed under DSS stimulation and restored following ASP intervention; concurrently, the classic anti-inflammatory cytokines IL-10 and TGF-β1 were also restored, while the pro-inflammatory cytokines TNF-α, IL-6, and IL-17 were downregulated. It is worth noting that recent reports have indicated that ultrasound-optimized plant flavonoids can alleviate DSS-induced colitis by reducing pro-inflammatory cytokine levels and restoring anti-inflammatory cytokine levels [53]. This is fully consistent with the cytokine expression patterns observed in this study. Furthermore, ASP supplementation rescued DSS-induced intestinal barrier damage by enhancing the expression of tight junction proteins, thereby preserving intestinal structural integrity. These findings validate the efficacy of ASP in alleviating inflammation and barrier damage, expanding the knowledge of adlay seed polyphenols, which previously focused primarily on their antioxidant effects.

It is worth noting that role of ASP in anti-colitis is closely related to its regulation of gut microbiota, gut microbiota is involved in maintaining intestinal barrier, regulating immune response, and producing metabolic products, which affect occurrence and development of intestinal inflammation [9]. As natural bioactive plant compounds, possess advantages such as anti-inflammatory and antioxidant properties and low toxicity. The intestinal anti-inflammatory capacity of plant polyphenols is primarily attributed to their modulation of gut microbial structure and restoration of intestinal microecological homeostasis [7]. Accumulating evidence has demonstrated that plant polyphenols regulate gut microbiota through multiple synergistic mechanisms. On the one hand, polyphenols selectively suppress the proliferation of pathogenic bacteria while promoting the growth of beneficial intestinal microbes, thereby optimizing overall microbial community composition. On the other hand, owing to their potent antioxidant properties, polyphenols efficiently eliminate intestinal ROS, alleviate oxidative stress-mediated microbial damage, and ultimately stabilize intestinal microecological balance [54]. Polyphenols regulate intestinal mucosal barrier to restrict bacterial movement and reduce intestinal inflammation. According to reports, it can be found that abundance of Bacillota (formerly Firmicutes) and Actinobacteria will significantly decrease, while abundance of Bacteroidota, Pseudomonadota, and Campylobacter will increase. Which will lead to a decrease in F/B ratio, which is a symbol of intestinal microbiota imbalance [55]. ASP processing reverses this trend, which supports ASP’s ability to restore microbial balance and evaluate its potential.

At the genus level, remarkable alterations in gut microbial composition were observed in DSS-induced colitis mice relative to healthy controls. The Model group displayed significant enrichment of five bacterial genera, including *Escherichia-Shigella*, *Bacteroides*, *norank_f__Muribaculaceae*, *Allobaculum*, and *Dubosiella*, indicating that the abnormal proliferation of these genera is closely associated with the initiation and progression of intestinal inflammatory injury. In contrast, ASP intervention effectively restored gut microbial dysbiosis and improved microbial diversity in colitic mice. Specifically, the excessive abundances of *Escherichia-Shigella*, *Allobaculum*, *Bacteroides*, and *Dubosiella* were markedly downregulated after ASP treatment, with their levels gradually returning to the normal state of the Control group. Notably, *Escherichia-Shigella*, a Gram-negative opportunistic pathogen of the Enterobacteriaceae family, has been frequently reported to be highly enriched in patients with ulcerative colitis, which is recognized as a critical biomarker of intestinal microecological dysbiosis. [56]. In addition, toxic metabolites secreted by *Escherichia-Shigella* can directly injure intestinal epithelial cells, disassemble intercellular tight junctions and elevate intestinal permeability, thus forming a self-amplifying vicious cycle consisting of gut dysbiosis, mucosal injury and aggravated inflammatory response [9], thereby aggravating intestinal inflammation. Consistent with earlier investigations, our data revealed that this genus was over‑represented in colitic Model mice, and its abundance was effectively suppressed following ASP supplementation. The primary reason for the increased abundance is that the intestinal mucosal barrier in colitis-affected mice is compromised, leading to increased permeability and an imbalance in the intestinal microenvironment, which provides conditions for the overproliferation of *Escherichia-Shigella*. Concurrently, ROS and pro-inflammatory factors generated by intestinal inflammation further promote their growth and proliferation. The reduction in the abundance of this bacterial genus following ASP intervention may be achieved by inhibiting its proliferation and blocking the activation of inflammatory signaling pathways, thereby alleviating intestinal inflammation [57].

*Bacteroides* belongs to the Bacteroidota phylum and is a common commensal bacterium in the gut. Under normal conditions, it participates in nutrient metabolism and maintains microbial balance [50]. However, when it overproliferates, it can become an opportunistic pathogen. Studies have shown that excessive proliferation of *Bacteroides* can produce various proteases that degrade the MUC2 protein in the intestinal mucus layer, compromising the integrity of the mucosal barrier [43]. In this study, the overproliferation of *Bacteroides* in the Model group further exacerbated mucosal barrier damage and inflammatory responses. ASP treatment exerted a robust inhibitory effect on the proliferation of Bacteroides. The decreased abundance of Bacteroides lowered the levels of pro-inflammatory metabolites and proteases, thereby alleviating intestinal barrier damage, suppressing inflammatory cascades, and rebalancing the dysregulated gut microbiota. *Dubosiella* belongs to the Bacillota phylum, and its role in gut health remains controversial. In this study, excessive proliferation of *Dubosiella* in Model group can cause damage to intestinal mucosal barrier and expand inflammation mapping. After ASP intervention, its abundance will decrease, reducing damage to mucosal barrier and regulating intestinal resistance to alleviate inflammation mapping. This indicates a significant working mechanism by which ASP exerts anti-inflammatory effects. The aforementioned changes indicate that ASP reduces abundance of harmful bacteria such as *Escherichia-Shigella* and *Bacteroides* to alleviate intestinal inflammation, block toxin release, and activate inflammatory signals. Simultaneously increasing abundance of beneficial bacteria such as *Lachnospiraceae-NK4A136-group* to repair intestinal mucosal barrier. Suppressing vicious cycle between inflammatory response, biological imbalance, and intestinal inflammation, alleviating effect of ASP on colitis is closely related to its regulatory effect on intestinal microbiota. Summary of PICRUSt function prediction indicates that ASP has potential ability to regulate gut microbiota function in multiple dimensions: on the one hand, it restores the function of pathways related to basal metabolism and epithelial repair, thereby reestablishing the homeostasis of the intestinal microenvironment; on the other hand, it suppresses the activation of pathways associated with microbiota pathogenicity, reducing pro-inflammatory stimuli and host immune activation. These findings provide key functional-level evidence for the mechanism by which ASP improves ulcerative colitis.

The transcriptome − based differential gene enrichment analysis was then used to identify key pathways associated with ASP’s anti-colitis effects, including the PI3K-Akt signaling pathway, TNF signaling pathway, and phagocytic cytokine-cytokine receptor exchange efficacy. Integrated transcriptomic analysis indicated that PI3K Akt signaling pathway is crucial for resolution of ASP mediated colitis. PI3K-Akt channel plays a significant role in various biological stages, regulating several major downstream events in pathogenesis of UC [58]. It can regulate apoptotic signaling network related to anti apoptotic protein Bcl-2 to cascade and regulate construction of cell survival, downstream mTOR signaling pathway regulates autophagy activity through research methods [59]. Our *in vivo* PCR measurements demonstrated that ASP suppressed the mRNA abundance of PI3K, Akt and mTOR, lending experimental support to the hypothesis that the PI3K‑Akt cascade serves as a central mediator for ASP‑mediated bioactivity. Beyond its canonical downstream targets, PI3K‑Akt signaling exhibits extensive crosstalk with major inflammatory axes, such as TNF, IL‑17, NF‑κB, and JAK‑STAT pathways [60]. In this study, ASP markedly decreased circulating concentrations of TNF‑α, IL‑6 and IL‑17. This phenotypic change aligns well with the suppressed activation of pro‑inflammatory TNF, NF‑κB and JAK‑STAT cascades triggered by PI3K‑Akt repression. Collectively, these observations suggest that PI3K‑Akt signaling modulates multiple downstream inflammatory transduction events upon ASP intervention.

In summary, this study identified the mechanism by which ASP alleviates colitis based on the consistency of multiple lines of independent evidence. In the researchers’ studies on the effects of ultrasonic-treated plant polysaccharides and flavonoids against DSS-induced colitis [61], [62], [63], this multilevel consistency among transcriptional changes, gene expression validation, microbiome remodeling, and histological/phenotypic improvements has been repeatedly confirmed. In summary, gut microbiome analysis, transcriptomic analysis, and qRT-PCR validation collectively provide corroborating evidence that ASP alleviates colitis. Nevertheless, this study did not validate the PI3K/Akt/mTOR signaling pathway at the protein level, which remains a clear direction for future research. Nevertheless, we cannot exclude the possibility that ASP exerts direct effects on these inflammatory routes; dedicated mechanistic assays are warranted to clarify this point in subsequent investigations.

## 5 Conclusion

The results of this study indicated that ultrasonic processing effectively improved the yield of phenolic compounds in ASP, especially kaempferol, ferulic acid, and coniferyl alcohol, thereby endowing ASP with enhanced anti-inflammatory bioactivity. In addition, ASP significantly slowed weight loss in mice with colitis, mitigated the increase in DAI scores, improved colonic shortening, and reduced pathological damage to colonic tissue. ASP could strengthen intestinal barrier integrity by increasing the abundance of colonic tight junction proteins. In addition, ASP intervention reversed the inflammatory imbalance in colitic mice via downregulating pro-inflammatory IL-17, IL-6, and TNF-α and upregulating anti-inflammatory IL-4, IL-10, and TGF-β1, ultimately exerting a protective effect on the inflamed intestinal tract. This indicates that it enhances intestinal barrier function and improves inflammatory conditions. 16S rRNA sequencing results showed that ASP treatment increased diversity of gut microbiota, promoted growth of beneficial bacteria (such as *Lachnospiraceae-NK4A136-group*), and inhibited proliferation of potential pathogenic bacteria such as *Escherichia-Shigella* and *Bacteroides*. Functional prediction analysis indicates that ASP restores functions related to microbial basal metabolism and epithelial cell repair, while also affecting functions related to microbial pathogenicity. Mechanistic studies indicate that ASP effectively inhibits the activation of PI3K-Akt signaling. Discovery provides scientific basis for development of adlay seed based natural medicines or functional foods for treatment of colitis. It should be noted that these effects represent the combined action of the ASP, in which polyphenols are the predominant identified bioactive constituents, and protein-level validation of the key signaling pathways will be addressed in future studies. This study points out several areas that need to be explored. Including evaluating comparative effects of ASP with other drugs and elucidating therapeutic mechanism of ASP. Future research should evaluate regulatory mechanism of ASP on gut microbiota through fecal microbiota transplantation.

## Author statement

This manuscript has not been published previously, and is not under consideration for publication elsewhere.

All authors confirm that they have made substantial contributions to the conception and design of the study, the acquisition, analysis and interpretation of experimental data.

All authors participated in drafting or critically revising the manuscript for important intellectual content.

All authors have read and approved the final revised version of the manuscript, and agree to be accountable for all aspects of the work to ensure that questions related to the accuracy or integrity of any part of the work are appropriately investigated and resolved.

No AI generative tools were used in the writing of this manuscript.

The authors declare that we have no known competing financial interests or personal relationships that could have appeared to influence the work reported in this paper.

## CRediT authorship contribution statement

**Yue Wu:** Writing – original draft, Visualization, Software, Data curation. **Changsheng Guo:** Writing – review & editing, Validation, Software. **Xiaoqiang Li:** Writing – review & editing, Methodology. **Jianjun Cheng:** Writing – review & editing, Writing – original draft, Supervision, Resources, Conceptualization. **Qingfeng Ban:** Writing – review & editing, Supervision, Formal analysis.

## Declaration of competing interest

The authors declare that they have no known competing financial interests or personal relationships that could have appeared to influence the work reported in this paper.

## Appendix A Supplementary data

Supplementary Data 1 Supplementary animal experimental methods.
