# Supplementary material for: Effects of ultrasound on the component, anti-inflammatory activity of adlay seed polyphenols and the mechanism of alleviating colitis
Source: Ultrason Sonochem. 2026 Oct 29;134:108143. doi: 10.1016/j.ultsonch.2026.108143 (PMC13755592; doi:10.1016/j.ultsonch.2026.108143)
Supplement: Supplementary file 1 [file mmc1.docx]

**Effects of ultrasound on the component, anti-inflammatory activity of adlay seed polyphenols and the mechanism of alleviating colitis**

Yue Wu, Changsheng Guo, Xiaoqiang Li, Jianjun Cheng^*^, Qingfeng Ban^*^

College of Food Science, Northeast Agricultural University, Harbin, Heilongjiang 150030, China.

*Corresponding author: Jianjun Cheng and Qingfeng Ban

Tel: +86-0451-55190007

E-mail addresses: jjcheng@neau.edu.cn (Jianjun Cheng) and qfban@neau.edu.cn (Qingfeng Ban)

**Supplementary materials**

**Supplementary Figure Captions**

**Figure S1.** Integral experimental design of ASP

**Supplementary Table Captions**

**Table S1.** Disease activity index (DAI) score criteria of mice

**Table S2.** Primer sequences used in quantitative real-time polymerase chain reaction (qRT-PCR) assays

**Figure S1.** Integral experimental design of ASP.

**Table S1.** Disease activity index (DAI) score criteria of mice.

| Weight loss% | Fecal character | Blood with fecal | Score |
| --- | --- | --- | --- |
| 0 | Shape and hard | None | 0 |
| 1-5 | Formed but fluffy and invisible | Blood with fecal | 1 |
| 6-10 | Not rigorous | A little blooding is visible | 2 |
| 11-15 | Mild diarrhea | Amount of blooding visible | 3 |
| >15 | Severe diarrhea | Visible rectal bleeding | 4 |

**Table S2.** Primer sequences used in quantitative real-time polymerase chain reaction (qRT-PCR) assays.

| **Genes** | **Primer** **sequences** |
| --- | --- |
| *GAPDH* | CCTCGTCCCGTAGACAAAATG |
|  | TGAGGTCAATGAAGGGGTCGT |
| *PI3K* | TCTCAACTCGCCTCATAGCAG |
|  | CTTGCTCTGGCACACAGTCAT |
| *Akt* | GCTCTTCTTCCACCTGTCTCG |
|  | CGCAGAATGTCTTCATAGTGGC |
| *TSC2* | CAGGTGTGCAGAAGGCAAAC |
|  | TCCACTGCATGCTCCTCAAG |
| *mTOR* | GCAACAACCTCCAGGATACACTAA |
|  | ACGGGCTGTGGTGGTAGACTTA |
| *p70S6k* | ACCTGTTGACAGCCCTGATG |
|  | CTGACAGGTGTTCGTGGACT |
| *4EBP1* | AAGGGATCTGCCCACCATTC |
|  | CAAACTGTGACTCTTCACCGC |
| *Beclin-1* | CAGGCGAAACCAGGAGAGAG |
|  | GTAGCTGTTCACTGTCGCCC |
| *LC3B* | AGATCCCAGTGATTATAGAGCGA |
|  | ACTTCGGAGATGGGAGTGGA |
